# Defective HIV-1 envelope gene promotes the evolution of the infectious strain through recombination in vitro

**DOI:** 10.1186/s12879-020-05288-w

**Published:** 2020-08-04

**Authors:** Huamian Wei, Danwei Yu, Xiuzhu Geng, Yuxian He

**Affiliations:** 1 NHC Key Laboratory of Systems Biology of Pathogens, Institute of Pathogen Biology, Chinese Academy of Medical Sciences & Peking Union Medical College, Beijing, P. R. China; 2 Center for AIDS Research, Chinese Academy of Medical Sciences & Peking Union Medical College, Beijing, P. R. China

**Keywords:** HIV-1, Recombination, Envelope, Evolution, Defective gene

## Abstract

**Background:**

HIV-1 produces defective mutants in the process of reproduction. The significance of the mutants has not been well investigated.

**Methods:**

The plasmids of wild type (HIV-1_NL4–3_) and Env-defective (HIV-1_SG3_^ΔEnv^) HIV-1 were co-transfected into HEK293T cells. The progeny virus was collected to infect MT4 cells. The *env* gene and near-full-length genome (NFLG) of HIV-1 were amplified and sequenced. The phylogenetic diversity, recombinant patterns and hotspots, and the functionality of HIV-1 Env were determined.

**Results:**

A total of 42 *env* genes and 8 NFLGs were successfully amplified and sequenced. Five types of recombinant patterns of *env* were identified and the same recombinant sites were detected in different patterns. The recombination hotspots were found distributing mainly in conservative regions of env. The recombination between genes of HIV-1_NL4–3_ and HIV-1_SG3_^Δenv^ increased the variety of viral quasispecies and resulted in progeny viruses with relative lower infectious ability than that of HIV_NL4–3_. The defective *env* genes as well as NFLG could be detected after 20 passages.

**Conclusion:**

The existence of the defective HIV-1 promotes the phylogenetic evolution of the virus, thus increasing the diversity of virus population. The role of defective genes may be converted from junk genes to useful materials and cannot be neglected in the study of HIV-1 reservoir.

## Background

Human immunodeficiency virus type 1 (HIV-1) remains a global threat to public health with an estimated 36.7 million peoples living with HIV-1 (http://www.unaids.org). The genetic diversity of HIV-1 has continued to increase, which poses an additional challenge to the treatment and prevention of HIV-1 infection [1]. HIV-1 diversity can be attributed to low fidelity of reverse transcriptase in the process of biosynthesis of double stranded DNA, as reflected in its high rate of mutation as well as recombination between different viral strains [2]. Therefore, a large proportion of HIV-1 strains may be defective due to the spontaneous passage of lethal mutations [3]. In long-term non-progressors, levels of HIV-1 defectiveness have been reported to be as high as 64% in accessory genes and 41% in env V3 region [4]. Astonishingly, defective proviruses accumulate rapidly during acute HIV-1 infection to make up over 93% of all proviruses, regardless of how early antiretroviral therapy (ART) is initiated [5]. Defective provirus mutants may still play a role in HIV-1 pathogenesis through recombination and rescue of drug resistance phenotypes, and viral recombination may take place with defective viral forms among the quasispecies to increase viral fitness and transmission capacity [6].

In contrast to the slow and steady change caused by mutation, recombination is a much more powerful evolutionary force. First, recombination facilitates the repair of viral genomes. Recombination can bypass Muller’s ratchet by recreating mutation free individuals from a population of mutants [7]. Second, recombination can both create and maintain genetic diversity in a population [8]. Third, recombination can speed adaptation by eliminating competition among beneficial mutations [9]. Recombination is a key mechanism that facilitates the persistence of virus with latent envelope genomic fragments in the productively infected cell population [10]. Compared with other genes of HIV-1, *env* gene is undoubtedly the most variable with higher rate of mutation, deletion, and insertion [11]. The Env glycoproteins are required when HIV-1 enters into target cells, and the diversity of the *env* gene has been shown to increase continuously and peaks at the onset of AIDS [12]. It is clear that antiviral drugs unlikely have effect on integrated viral DNA, and the efficiency of CRISPR/Cas9 gene editing technology for integrated HIV-1 DNA may also reduce because of the mutations on the defective virus [13]. Although the defective HIV-1 occupies a considerable proportion in infections, the significance of env-defective HIV-1 mutants has not been well investigated. In this study, the evolution of superinfection of env-defective and infectious wild type HIV-1 strains in long-term in vitro passages was investigated.

## Methods

### Plasmids

HIV-1 infectious clone pNL4–3 and *env*-defective clone pSG3^ΔEnv^ were obtained from the AIDS Research and Reference Reagent Program [14, 15]. pSG3^Δenv^ was derived from pSG3 (L02317) by the introduction of four nucleotides (CTAG) which generated a translational stop codon after amino acid residue 142 in the *env* gene. When the plasmid pSG3^Δenv^ was transfected into HEK 293 T cells alone, all proteins of HIV-1 excepted Env could be expressed functionally. If another plasmid expressing Env was co-transfected, the pesudovirus could be generated. The intact *env* genes of recombinant strains as well as NL4–3 and SG3 were amplified and cloned into pcDNA3.1 vector (Cat No.: K4900–01, Invitrogen) to construct Env expression vectors and to evaluate the infectious ability. Ethics approval was deemed unnecessary according to national regulations.

### Cell culture, transfection and infection

HEK293T cells purchased from ATCC were cultured in Dulbecco’s modified Eagle’s medium (DMEM) supplemented with 10% fetal bovine serum (FBS), 100 μg/mL streptomycin and 100 IU/mL penicillin. The pSG3^Δenv^ and pNL4–3 were co-transfected into HEK 293 T cells. After 8 h (h) of transfection, the medium was discarded and the cells were washed twice gently with phosphate buffer saline (PBS), followed by adding fresh DMEM completed medium. The cells were cultured for another 36 to 48 h, and virus supernatant was collected. MT4 cells (obtained through the NIH AIDS Reagent Program, originally acquired from Dr. Douglas Richman) were seeded on 12-well culture plates at 1 × 10^5^ cells per well with the RPMI 1640 medium containing 10% FBS and incubated with the virus supernatant for 2 h, then washed twice with PBS and resuspended with the RPMI 1640 complete medium. The cells were incubated at 37 °C with 5% CO_2_, and the medium was half changed every 3 days until an extensive cytopathic effect (CPE) was observed. Then, the progeny virus was passaged on MT4 cells in four duplicate wells (Supplemental Fig. S1). As controls, the pSG3^Δenv^ and pNL4–3 were respectively transfected into HEK 293 T cells. To eliminate the potential effect of plasmid contamination, the HEK 293 T cells were transfected with pSG3^ΔEnv^, pNL4–3, pSG3^ΔEnv^ + pNL4–3, and pcDNA3.1, respectively. After 48 h, the cells and the supernatant were collected. 500 μl of the virus supernatant or inactivated virus (100 °C for 10 min) was in parallel used to infect the MT4 cells. After 48 h, the MT4 cells were collected. The genome DNA of the cells from each group was extracted. The *env* gene was amplified. The PCR gel electrophoresis was carried out to identify the positive band of the *env* gene. The MT4 cells and the supernatant of each passage were collected and stored at − 80 °C for subsequent assays.

### Amplification of *env* gene and near-full-length genome (NFLG)

The provirus DNA was extracted from the MT4 cells by DNeasy Blood & Tissue Kit (Cat No.: 69504, QIAGEN). The extracted DNA was properly diluted to avoid PCR-induced recombination. The fold of optimum dilution (D) was estimated according to the formula: $\mathrm{D}=\frac{\left(\mathrm{N}1/\mathrm{R}\right)\times \mathrm{D}1+\left(\mathrm{N}2/\mathrm{R}\right)\times \mathrm{D}2+\left(\mathrm{N}3/\mathrm{R}\right)\times \mathrm{D}3}{\mathrm{NR}}\div 25\%$, where D1, D2 and D3 were dilution gradients, N1, N2 and N3 were the number of positive bands in each gradient, R was the number of duplicate wells for each gradient and NR was the number of positive gradient in which at least one positive band was observed. For *env*, the fold of dilution carried out in the study was initially set to 120, but for NFLG, we didn’t perform the dilution due to the lower amplification efficiency. The *env* gene was amplified by nested polymerase chain reaction (nest-PCR) with the first round primers H1OF (5′- TAGAGCCTTGGAAGCATCCAGGAAGTCAG-3′, 5583–5881, refer to HXB2: K03455) and H1OR (5′- CTCCATGTTTTTCCAGGTCTCGAGAT -3′, 8920–8895) and the second round primers H1IF (5′-CACCAAAGGCTTAGGCATCTCCCATGGCAGGAAGAAG-3′, 5947–5983) and H1IR (5′- TCCCACCCCATCTGCTGCTGGCTCAG-3′, 8889–8864). The PCR amplification conditions were conducted with one cycle of pre-denaturation at 94 °C for 5 min, followed by 35 cycles of denaturation at 94 °C for 30 s, annealing at 60 °C for 30 s and extension at 72 °C for 3 min, and with a final extension at 72 °C for 10 min. For Env expression vector construction, the primers Env-F (5′-CAAGCTTGACAGTGGCAATGAGAGTGAAGGAG-3′, 6215–6239) and Env-R (5′- GCTCTAGAATACTGCTCCCACCCCATCTGCTG-3′, 8896–8873) were used as the second round primers with the same amplification condition. The amplified env gene was inserted into the pcDNA3.1 vector through the nucleotide endonuclease HindIII and XbaI. The near-full-length genome amplification was conducted as previously described [16]. The first round PCR was carried out using the primers FL1.5 (5′-CCTTGAGTGCTTCAAGTAGTGTGTGCCCGTCTGT-3′, 538–571) and FL1.3 (5′-ACTACTTGAAGCACTCAAGGCAAGCTTTATTG-3′, 9642–9611), and the second round primers FL2.5 (5′-AGTAGTGTGTGCCCGTCTGTTGTGTGACTC-3′ 552–581) and FL2.3 (5′- TGAAGCACTCAAGGCAAGCTTTATTGAGGC -3′, 9636–9607). The PCR thermo-cycling conditions were as follows: one cycle of pre-denaturation at 94 °C for 2 min; 10 cycles of a denaturing step at 94 °C for 10 s and an extension step at 68 °C for 8.5 min; 20 cycles of denaturation at 94 °C for 10 s and extension at 68 °C for 8.5 min with an incremental of 20 s for each successive cycle; a final cycle of extension at 68 °C for 20 min.

### Sequence analysis

The positive PCR products were purified and sequenced by the cycle sequencing and dye terminator methods on an ABI 3730xl genetic analyzer (Applied Biosystems, Foster City, CA). Individual sequences were assembled and edited using Sequencher v4.9 (Gene Codes, Ann Arbor, MI). The sequences were aligned using CLUSTAL W, and the manual adjustment for optimal alignment was performed using BioEdit. Phylogenetic analysis was performed to determine the evolution of HIV-1 provirus with the reference sequences of NL4–3 and SG3. The maximum likelihood (ML) tree was constructed using the general time reversible (GTR) plus gamma model by PhyML [17]. Branches with bootstrap values higher than 0.9 were considered as phylogenetic clusters. Branch significance was analyzed with 200 bootstrap replicates. Sequences that cannot be clustered into NL4–3 or SG3 group were further verified by bootscan analysis using Simplot [18] with window size of 180 bp and step size of 10 bp for *env* genes, and with window size of 300 bp and step size of 30 bp for NFLGs. The recombinant breakpoints were identified and the recombinant sequences were mapped by Recombinant HIV-1 Drawing Tool (https://www.hiv.lanl.gov/content/sequence/DRAW_CRF/recom_mapper.html). The possible hotspots of recombination across env gene was evaluated by RAPR [19] (https://www.hiv.lanl.gov/content/sequence/RAP2017/rap.html).

### Single cycle infection assay

The infectious ability mediated by HIV-1 recombinant Env protein was measured by a single-cycle infection assay as described previously [20]. Briefly, HIV-1 pseudovirus was generated by co-transfecting 293 T cells with an Env-expressing plasmid and a backbone plasmid pSG3^Δenv^. The supernatant was harvested 48 h after transfection, and 50% tissue culture infectious dose (TCID_50_) was determined using TZM-bl cells (NIH AIDS Reagent Program). The infectious ability of recombinant strains was determined by infection of TZM-bl cells with 100 TCID_50_ virus dose. The cells were incubated for 48 h at 37 °C, and the luciferase activity (relative light unit, RLU) was measured using luciferase assay reagents (Cat No.: G7941, Promega) and a luminescence counter GloMax (Promega). Student *t* test was performed to compared the difference. *p* value less than 0.05 was considered significant statistical difference.

## Results

### Diversity and phylogenetic analysis of HIV-1 provirus

**Fig. 1:**
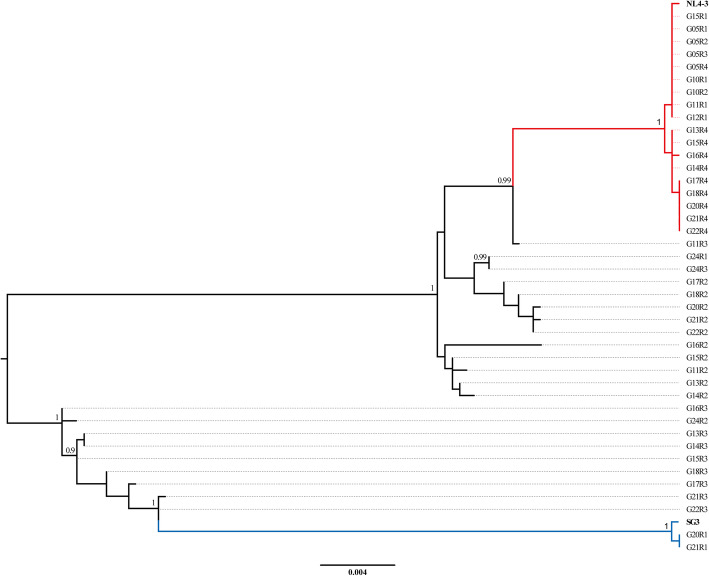
Construction of phylogenetic tree. The parent strains NL4–3 and SG3 were bold marked. Branches with bootstrap values higher than 0.9 were considered as phylogenetic clusters. The NL4–3 and SG3 clusters were respectively colored in red and blue. The sequences in the two parent clusters were recognized as pure strains and those between them were considered as recombinants

The plasmids pSG3^Δenv^ and pNL4–3 were co-transfected into HEK 293 T cells and the progeny virus was used to infect MT4 cells. Since the recombination could have occurred during co-transfection or during viral replication in MT4 cells, we conducted single genome amplification with the genome DNA from the co-transfected HEK 293 T cells. Phylogenetic analysis revealed that there was no recombination between NL4–3 and SG3 (Supplemental Fig. S2). Meanwhile, to eliminate the possibility of plasmid contamination, the inactivated supernatant was used to infect the MT4 cells. The corresponding genome DNA of MT4 cells was extracted and the *env* gene was amplified (Supplemental Fig. S3). It was found that the *env* gene was successfully amplified in the transfected HEK 293 T cells as well as the MT4 cells infected with the non-inactivated supernatant. As a control, no positive band was found in the MT4 cells infected with the inactivated supernatant. A total of 42 HIV-1 *env* genes were successfully amplified and sequenced from the cell genomes of the 5th to 24th passages (GenBank: MG837222 - MG837263). An initial alignment was performed using Clustal W, and then adjusted manually using BioEdit. Phylogenetic tree analyses were implemented by PhyML [17] and branches with bootstrap values higher than 0.9 were considered as phylogenetic clusters (Fig. 1). The parent strains NL4–3 and SG3 were bold marked. As the phylogenetic tree displayed, the sequences could be divided into several clusters. There were 18 sequences grouped into the NL4–3 cluster, in which the genetic distance of all the sequences was extremely small. Unexpectedly, two sequences amplified in the 20th and 21st passages were grouped into the SG3 cluster. The alignment of the three sequences found that the two sequences in the clade were almost the same with SG3, except for an insertion of four-nucleotides after the position of 423 T, which was the characteristic of pSG3^ΔEnv^*env* gene. Besides the NL4–3 and SG3 clusters, there were 22 sequences distributed between the two clusters indicating the emergence of recombination between HIV_NL4–3_ and HIV_SG3_^Δenv^.

### Determination of recombinant patterns and breakpoints

**Fig. 2:**
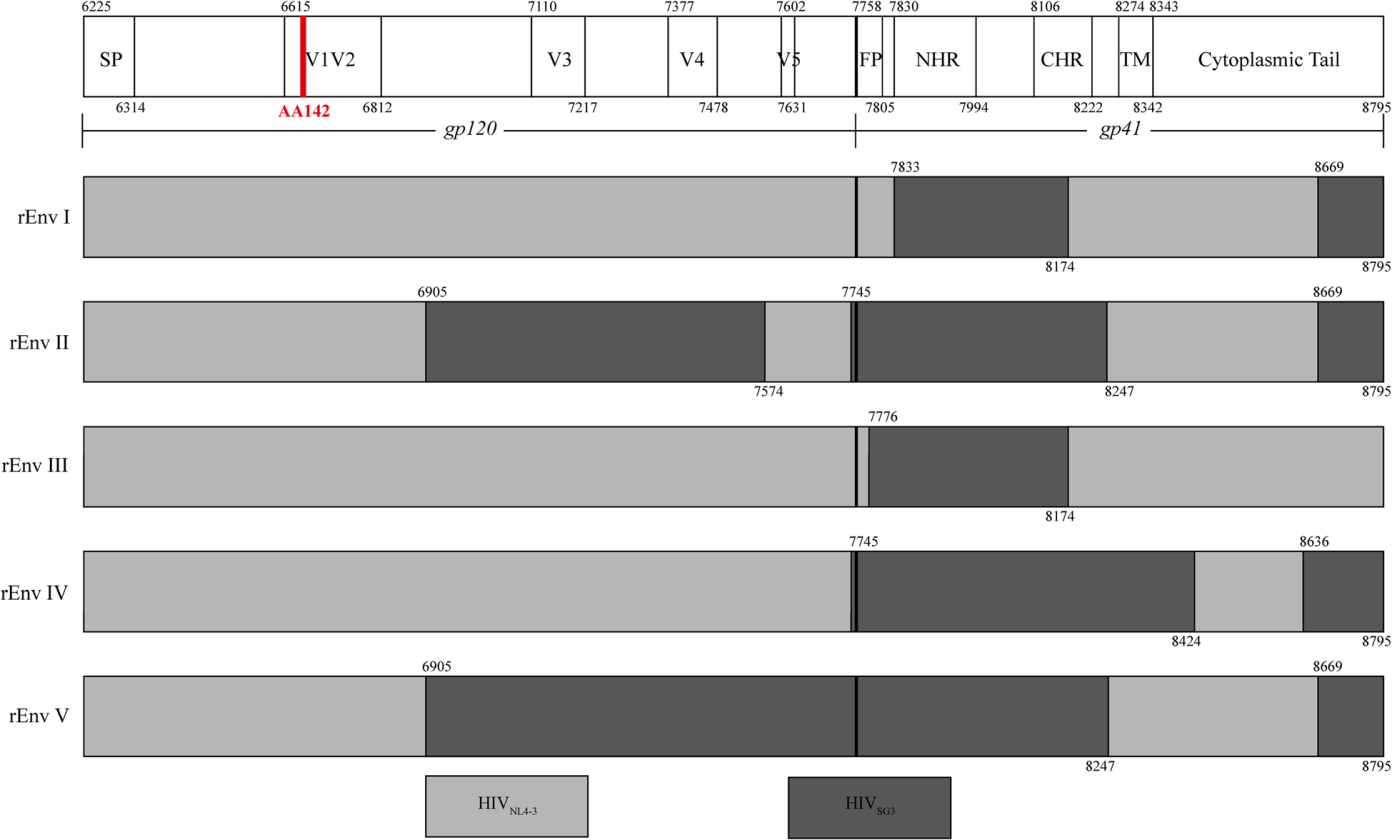
Determination of recombinant patterns and breakpoints. The *env* genes of parent strains NL4–3 and SG3 as well as a CRF01_AE strain were used as reference sequences when performing the bootscan analysis with window size of 180 bp and step size of 10 bp using Simplot software. Position numbering is relative to the genome of HXB2. The AA142 position was red marked indicating where the nucleotide acids CTAG inserted. SP, signal peptide. FP, fusion peptide. NHR, N-terminal helix repeat region. CHR, C-terminal helix repeat region. TM, Transmembrane domain

**Fig. 3:**
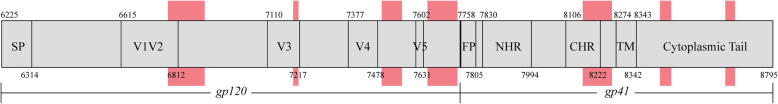
Identification of the significant hotspots in *env*. Recombination hotspots were shown in red across the *env* gene. Each line represented a position, and the thickness of the colored regions represents consecutive positions. Position numbering is relative to HXB2. SP, signal peptide. FP, fusion peptide. NHR, N-terminal helix repeat region. CHR, C-terminal helix repeat region. TM, Transmembrane domain

The potential recombinant sequences between the parent clusters were verified by bootscan analysis, and the recombinant and breakpoints were identified using Simplot [18]. The *env* genes of NL4–3 and SG3 were used as the parent reference sequences and a CRF01_AE strain (AY008714) was used as an outgroup reference sequence. All *env* genes were evaluated and 5 types of recombinant patterns named rEnvI, rEnvII, rEnvIII, rEnvIV and rEnvV were identified (Fig. 2). Among them, rEnvIand rEnvII detected in different passages were the main recombinant patterns, which cover 81.8% (18/22) of the sequences. The different recombinant patterns indicated that either the replacement of the corresponding recombinant fragments of NL4–3 with that of SG3^Δenv^ or the repair of the SG3^Δenv^ defective with the normal NL4–3 genome. Furthermore, there were several identical breakpoints were found between different recombinant patterns, such as the position 6905, position 7745, position 8174, position 8247 and position 8669 (refer to HXB2). These recombinant strains were detected from different duplicate wells. The similar recombinant sites found in different passages from the same well might be due to the expansion of the recombinant, but that from the different wells represented higher possibility of recombination in this region, and this region might be a hot area of recombination. Thus, it was inferred that the same breakpoints in the recombinant strains suggested the existence of recombination hotspots. The significance of each breakpoint in the study was revalued by RAPR [19] and the hotspots of recombination across the env gene were calculated (Fig. 3). It was found that hotspots were mostly clustered in relatively conservative regions after the variable loops of gp120 or the C-terminal helical repeat region and cytoplasmic tail region of gp41.

### Env-mediated HIV-1 infectivity

**Fig. 4:**
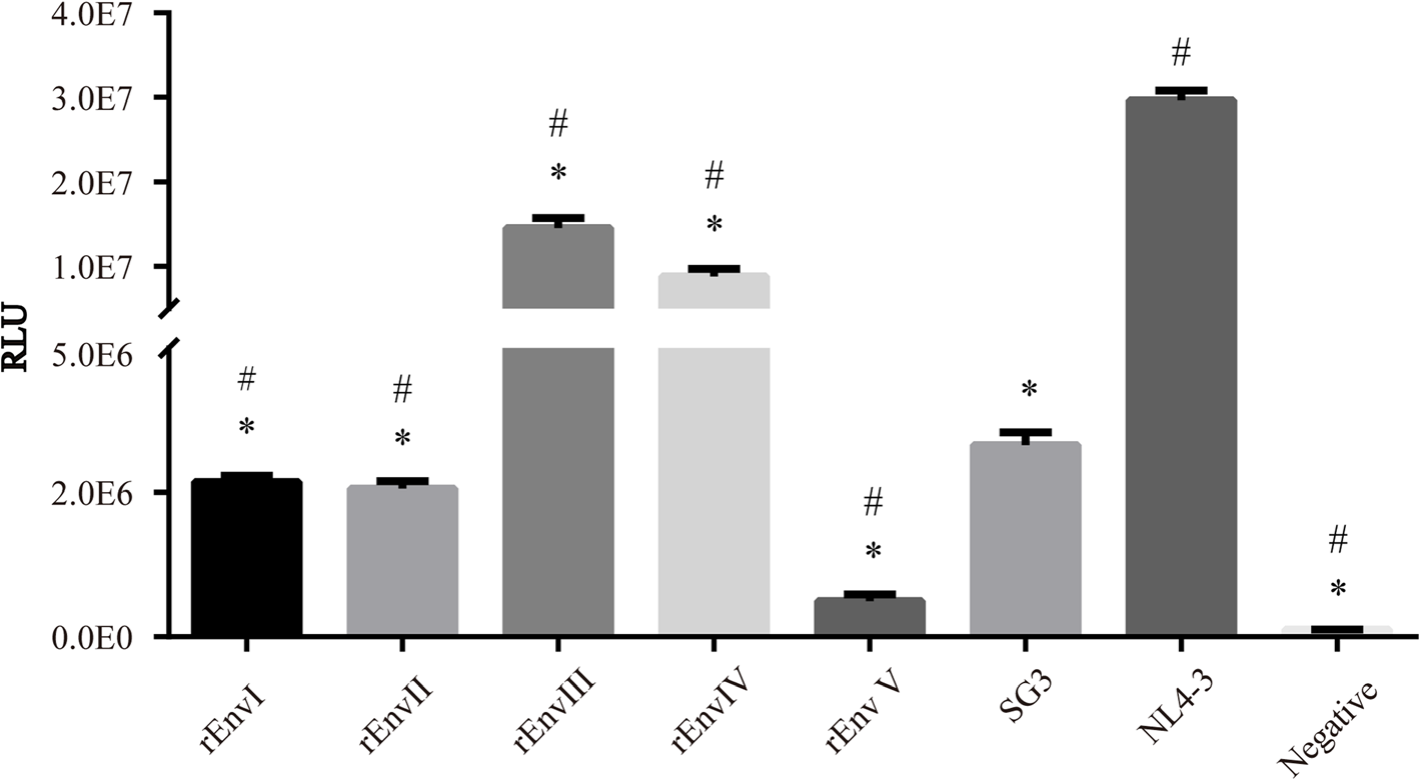
Env-mediated HIV-1 infectivity. The pseudoviruses packed with Env recombinants and the parent strains NL4–3 and SG3 were generated and the infectious ability was measured by a single-cycle infection assay. RLU, relative light unit. *, *p* < 0.05 when compared with NL4–3. #, *p* < 0.05 when compared with SG3

The *env* gene of each recombinant pattern was amplified and merged into the pcDNA3.1 expression vector to construct recombinant Env expression vectors. The infectious ability mediated by recombinant Env was determined and compared with that of the wild-types NL4–3 and SG3. Pseudoviruses were generated using pSG3^Δenv^ as the package vector. 100 TCID_50_ of viruses were used to infect TZM-bl cells. It was found that all expressed recombinant Envs were functional, and the Env-mediated infectivity was significantly different between recombinant and parent strains. Compared with the HIV_NL4–3_, all other strains showed lower infectious ability, especially the rEnvV (all *p* < 0.05). However, the infectious ability of rEnvIII and rEnvIV was markedly increased compared to HIV_SG3_ (all *p* < 0.05). The results demonstrated that the infectious HIV-1 strain could alter its biological characteristic by recombining its own gene fragment with the intact part of a defective virus (Fig. 4).

### Recombination of HIV-1 near-full-length genome

**Fig. 5:**
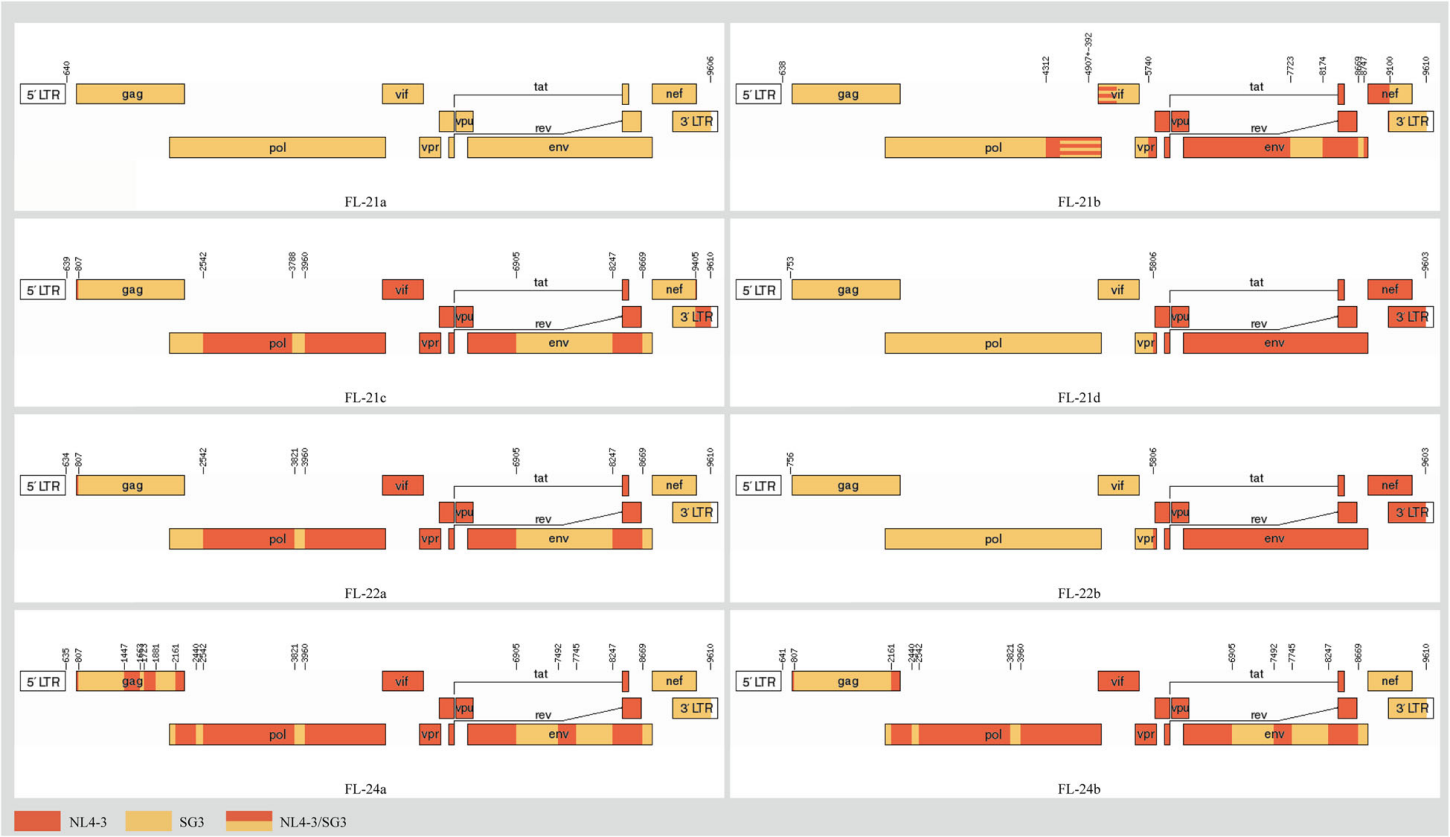
Recombination of HIV-1 near-full-length genome. The genomes of parent strains NL4–3 and SG3 as well as a CRF01_AE strain were used as reference sequences when performing the bootscan analysis with window size of 300 bp and step size of 30 bp using Simplot software. Position numbering is relative to the genome of HXB2. The recombinant map was drawn by Recombinant HIV-1 Drawing tool in Los Alamos HIV Sequence Database

Since the recombination positions might have occurred anywhere of HIV genome, the near-full-length genome (NFLG) was amplified to further understand the recombination of HIV_NL4–3_ and HIV_SG3_^Δenv^. A total of 8 NFLGs were amplified and sequenced from the cell genome of the 21st, 22nd and 24th passages (GenBank: MG837264 - MG837271). The recombinant patters and breakpoints of the NFLG sequences were identified (Fig. 5). In all the NFLGs, there were 7 recombinants and 1 pure subtype stain. FL-21d and FL-22b share the identical recombinant pattern and breakpoints, and the recombinant patterns and breakpoints between FL-21c and FL-22a, as well as FL24-a and FL24-b were almost the same but with some difference in the end of 3’LTR and gag regions, respectively. The same recombination sites could be found between different recombinant strains, as exemplified by the positions 2542, 3960, 6905, 8427and 8669.

### Long term persistence of defective HIV-1

**Fig. 6:**
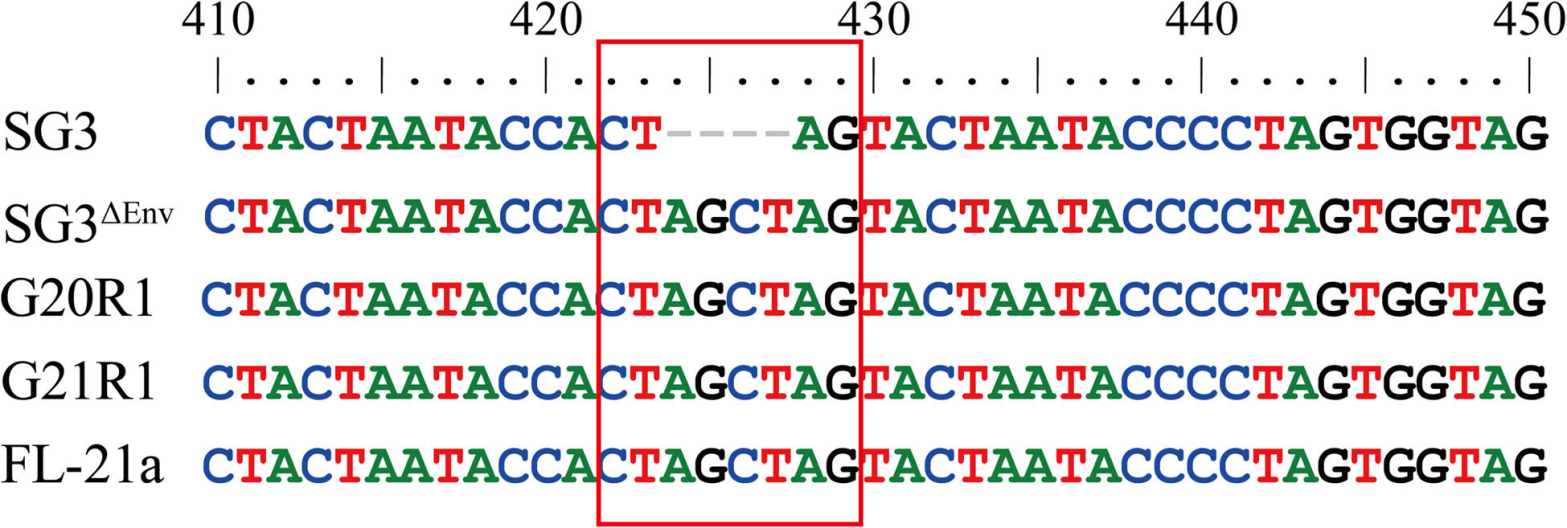
Identification of *env*-defective gene after 20th passage. The *env*-defective sequences G20R1 and G21R1 as well as the NFLG sequence FL-21a were aligned with the sequence of SG3 and SG3^Δenv^. The characteristic of the 4-nucleoid acid (AGCT) insertion after position 423 T was identified in the sequences isolated from the 20th and 21st passages in the host cell genome. Position numbering is relative to the *env* of SG3

Besides the recombinant strains, pure *env* sequences G20R1 and G21R1 and near-full-length genome FL-21a with an identical nucleotide acid sequence of HIV_SG3_^Δenv^ were determined from HIV-1 provirus genome. By alignment with the mask sequence of SG3 and SG3^ΔEnv^, an insertion of four nucleoid acids (AGCT) after T at the position 423, which was identical with the sequence of SG3^ΔEnv^ (Fig. 6). Considering that the pure sequences were amplified from the genomes of the 20th and 21st passages, it was ascertained that the defective gene of HIV-1 might persist and passage in the host cell genome with the help of infectious strains.

## Discussion

HIV-1 displays in the form of *quasispecies* which is one of the hallmarks of HIV-1 infection [21, 22]. Previous studies demonstrate that a single viral particle can lead to infection [23, 24]. During the HIV-1 replication, the rate of nucleotide misincorporation was 3.4 × 10^− 5^/base/cycle [25]. With the high rate of mutation, the defective viruses can rapidly accumulate during acute HIV-1 infection and continue to increase as the process of the disease [4, 5]. Even though the defective virus exists in the whole life cycle of HIV-1 infection, its effects on evolution, fitness and disease progression are rarely studied because of its non-infectious characteristic. It has been reported that morn then 1 HIV copy is found in infected spleen cells; as well, a single cell can harbor several different copies of HIV-1 NDA [26]. Therefore, cells contain defective HIV-1 may still produce defective viral particles. Moreover, it has been revealed that HIV-1 infected cells with 5 copies of defective provirus are able to generate highly infectious viral progeny [27]. In this study, co-transfection of the plasmids of the Env-defective virus HIV_SG3_^Δenv^ and the infectious virus HIV_NL4–3_ in HEK 293 T cells resulted in a large number of recombinant progeny strains. The recombination between genes of HIV_NL4–3_ and HIV_SG3_^Δenv^ increased the variety of the infectious HIV-1 strain, and the variation of HIV_NL4–3_ or HIV_SG3_^Δenv^ was promoted by replacing its genome fragments with that of HIV_SG3_^Δenv^ or HIV_NL4–3,_ respectively.

HIV-1 superinfection can occur at any stage of the disease process despite the preexisting host immune response to the initial virus and rates of superinfection have been estimated to be close to the rates of initial infection, indicating a lack of protective immunity against newly acquired HIV-1 infection by preexisting infection [28–30]. However, superinfection may be difficult to be detected when the superinfecting virus is of the same subtype as the initial virus, and recombination between these viruses is often ignored. In the study, phylogenetic analysis and bootscan breakpoint analysis were performed using HIV_NL4–3_ and HIV_SG3_ as parent strains, and the recombinant *env* genes were firstly detected in the 11th progeny virus infected cells. By analyzing recombinant pattern and breakpoint of *env* genes, it was found that the same recombinant sites appeared in different recombination patterns with one to three gene fragments replacement, implying the possibility of a second or multiple recombination. Indeed, Simon-Loriere and coworkers identified the same pattern [31]. Due to the limited sequences amplified, the bias of recombinant hotspots might exist. However, when compared with the recombination sites identified by bootscan analysis, the results are consistent, where most of the recombination breakpoints are in the recombinant hotspots. Furthermore, recombination in the other regions of HIV-1 genome was also observed. Thus defective virus resulted from gene mutation, deletion and insertion may promote the evolution of replication-competent HIV-1 by superinfection or coinfection.

Previous studies suggest an association between HIV-1 fitness, diversity, recombination, rate of transmission, and disease progression [32, 33]. The very fit viruses have to adapt to a given environment in order to survive. The most fit virus in an ex vivo culture suggests an increased virulence in a host. However, rapid disease progression is also related to faster extinction of this viral isolate in the human population [34]. Ex vivo fitness of primary HIV-1 isolates typically maps to the *env* gene and is largely controlled by the efficiency of host cell entry [35]. It was shown that the recombinant Env proteins presented various infectious abilities. Compared with the HIV-1_NL4–3_ strain, the fitness of all other viruses was lower, especially the rEnvV. However, the infectious ability of rEnvIII and rEnvIV was significantly increased compared to that of HIV-1_SG3._ HIV-1_NL4–3_ is an ex vivo fitness strain, and the nucleotide acid of *env* gene is the result of an ex vivo culture adaptation. The replacement with *env* gene of HIV-1_SG3_^ΔEnv^ results in a large number of mutations. Therefore, the decline of fitness of recombinant strains is predictable.

Highly active antiretroviral therapy (HAART) can effectively inhibit HIV-1 in the patients, but due to the high variation of the virus, the emergence and epidemic of drug resistant strains have become a serious problem that has to be faced. Meanwhile, the patient must take the drug for whole life in that the virus will proliferate again because of the persistence of a small reservoir of infected cells. It is reported that defective genomes were systematically detected in all patients on long-term HAART in both PBMCs and rectal tissues, and a high level of defective genomes was correlated with a small size of HIV-1 provirus DNA [36]. Furthermore, latent HIV-1 can be activated by exosomes from cells infected defective HIV-1 [37]. In the present study, two *env* sequences and one NFLG with the characteristic inserted fragment of HIV_SG3_^Δenv^ were identified after 20 passages, suggesting that the defective HIV-1 could persist in the host and passage with the help of infectious one and served as a kind of latent HIV-1. The persistence of HIV-1 reservoir has been one of the obstacles to eradicate HIV-1 infection. The Shock/Kick and Kill strategy and CRISPR/Cas9 gene editing technology play an important role in eradicating the HIV-1 reservoir [38–40]. Nevertheless, the coinfection or superinfection of defective and functional HIV-1 and high rate of recombination between them put forward a higher requirement for the elimination of the HIV-1 reservoir.

## Conclusion

The evolution of HIV-1 in the host is complex and subject to the pressure of the immune system. Defective viruses are produced in the process of continuous evolution of HIV-1. However, the role of those defective genes might be converted from junk genes to useful materials as the immune status changed. Defective species can potentially be a part of the HIV-1 reservoir and may contribute over time to fully infectious viral progeny through recombination. Therefore, the existence of the defective HIV-1 promotes the evolution of the virus, increases the diversity of HIV-1 population, and to a certain extent, may affect the immunization effect and the clearance of the HIV-1 reservoir.

## Supplementary information

**Additional file 1: Figure S1.** The diagram of the experimental design.

**Additional file 2: Figure S2.** Recombination identification of the proviruses in co-transfected HEK 293 T cells. The genome DNA of HEK 293 T cells co-transfected with pNL4–3 and pSG3ΔEnv was extracted and the single genome amplification was performed. A total of 40 sequences were obtained and subsequent for phylogenetic analysis to investigate whether there were recombinant proviruses. The evolutionary history was inferred using the Neighbor-Joining method. The percentage of replicate trees in which the associated taxa clustered together in the bootstrap test (1000 replicates) are shown next to the branches. The tree is drawn to scale, with branch lengths in the same units as those of the evolutionary distances used to infer the phylogenetic tree. The evolutionary distances were computed using the Kimura 2-parameter method and are in the units of the number of base substitutions per site. The analysis involved 42 nucleotide sequences. Codon positions included were 1st + 2nd + 3rd + Noncoding. All ambiguous positions were removed for each sequence pair. There were a total of 2619 positions in the final dataset. Evolutionary analyses were conducted in MEGA7.

**Additional file 3: Figure S3.** The *env* gene amplification. The HEK 293 T cells transfected with pSG3ΔEnv, pNL4–3, pSG3ΔEnv + pNL4–3 and pcDNA3.1 respectively. After 48 h, the cells and the supernatant were collected. Partial of the supernatant was inactived at 100 °C for 10 min. Then the equal volume (500 μl) of the fresh supernatant and the inactived one was used to infect the MT4 cells. After 48 h, the MT4 cells were collected. The genome DNA of the cells from each group was extracted. The env gene was amplified. The PCR gel electrophoresis was carried out to identify the positive band (red box). NC, negative control, transfected with pcDNA3.1 or infected with the supernatant from the NC group.

## Data Availability

The sequences obtained in this study were submitted to NCBI GenBank under Accession Numbers MG837222 - MG837271.

## Abbreviations

ART: antiretroviral therapy
CPE: cytopathic effect (CPE)
DMEM: Dulbecco’s modified Eagle’s medium
FBS: fetal bovine serum
GTR: general time reversible
HAART: highly active antiretroviral therapy
HIV-1: human immunodeficiency virus type 1
ML: maximum likelihood
NFLG: near full-length genome
PBS: phosphate buffer saline
PCR: polymerase chain reaction
RLU: relative light unit
TCID50: 50% tissue culture infectious dose
