# Supplementary figures and images for: Defective HIV-1 envelope gene promotes the evolution of the infectious strain through recombination in vitro
Source: BMC Infect Dis. 2020 Aug 4;20:569. doi: 10.1186/s12879-020-05288-w (PMC7401196; doi:10.1186/s12879-020-05288-w)

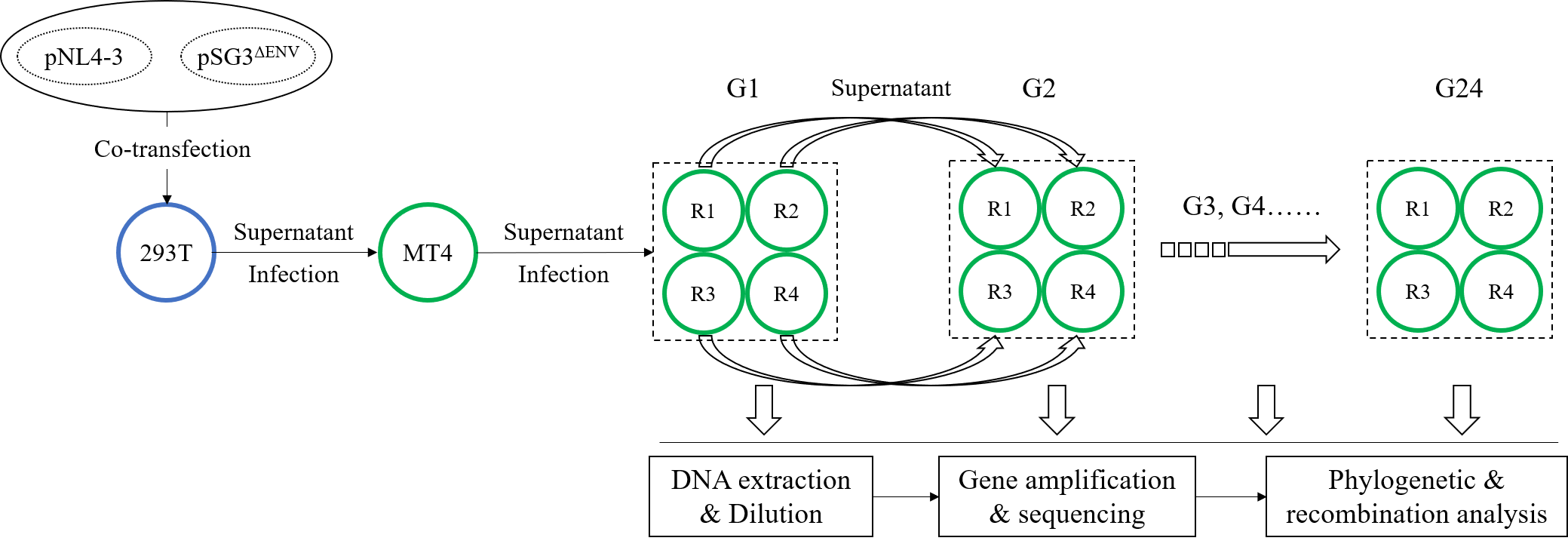

Supplement: Supplementary file 1 — Additional file 1: Figure S1. The diagram of the experimental design. [file 12879_2020_5288_MOESM1_ESM.tif]

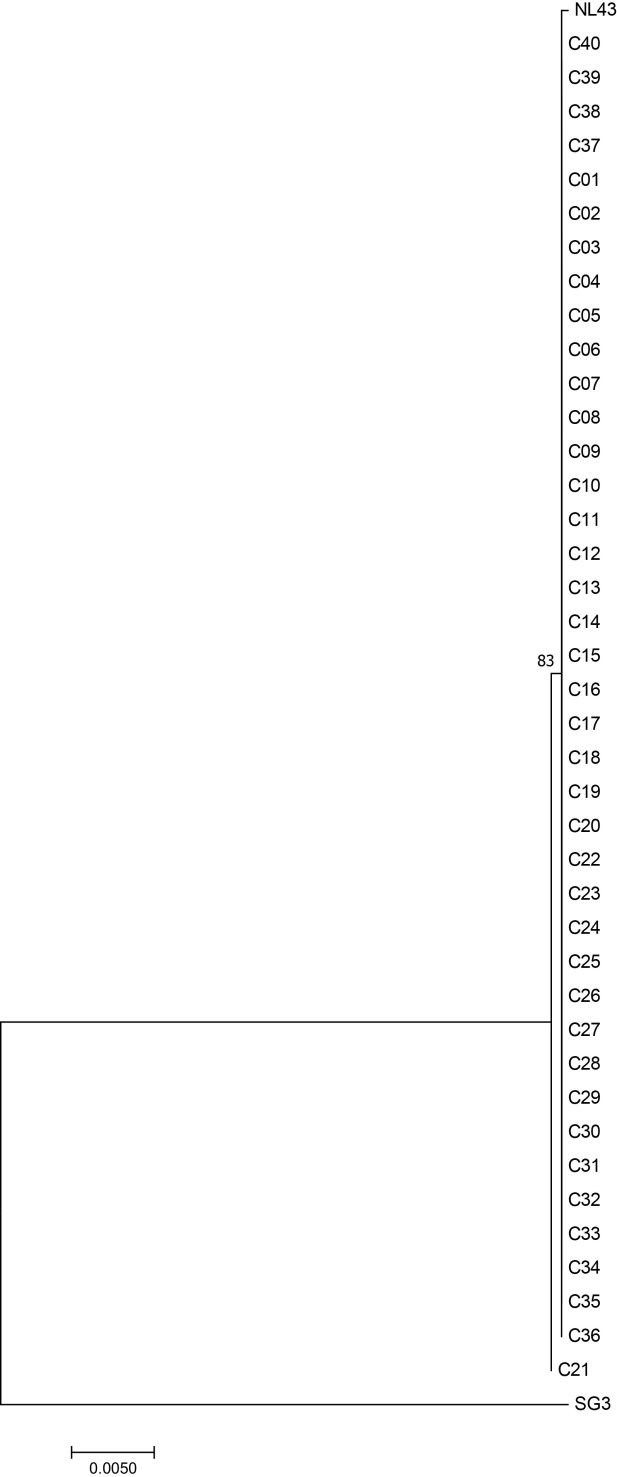

Supplement: Supplementary file 2 — Additional file 2: Figure S2. Recombination identification of the proviruses in co-transfected HEK 293 T cells. The genome DNA of HEK 293 T cells co-transfected with pNL4–3 and pSG3ΔEnv was extracted and the single genome amplification was performed. A total of 40 sequences were obtained and subsequent for phylogenetic analysis to investigate whether there were recombinant proviruses. The evolutionary history was inferred using the Neighbor-Joining method. The percentage of replicate trees in which the associated taxa clustered together in the bootstrap test (1000 replicates) are shown next to the branches. The tree is drawn to scale, with branch lengths in the same units as those of the evolutionary distances used to infer the phylogenetic tree. The evolutionary distances were computed using the Kimura 2-parameter method and are in the units of the number of base substitutions per site. The analysis involved 42 nucleotide sequences. Codon positions included were 1st + 2nd + 3rd + Noncoding. All ambiguous positions were removed for each sequence pair. There were a total of 2619 positions in the final dataset. Evolutionary analyses were conducted in MEGA7. [file 12879_2020_5288_MOESM2_ESM.tif]

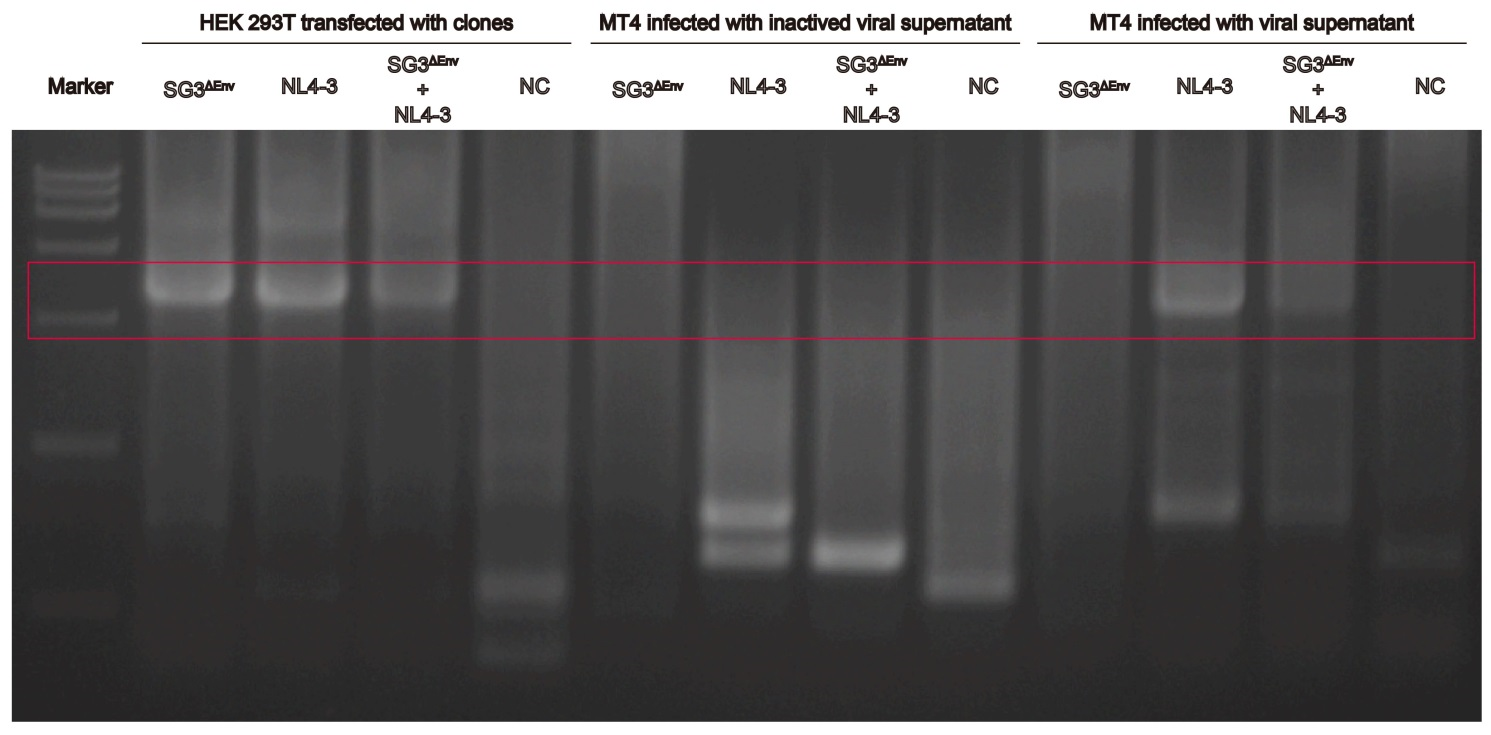

Supplement: Supplementary file 3 — Additional file 3: Figure S3. The env gene amplification. The HEK 293 T cells transfected with pSG3ΔEnv, pNL4–3, pSG3ΔEnv + pNL4–3 and pcDNA3.1 respectively. After 48 h, the cells and the supernatant were collected. Partial of the supernatant was inactived at 100 °C for 10 min. Then the equal volume (500 μl) of the fresh supernatant and the inactived one was used to infect the MT4 cells. After 48 h, the MT4 cells were collected. The genome DNA of the cells from each group was extracted. The env gene was amplified. The PCR gel electrophoresis was carried out to identify the positive band (red box). NC, negative control, transfected with pcDNA3.1 or infected with the supernatant from the NC group. [file 12879_2020_5288_MOESM3_ESM.tif]
